# Changes in metabolic phenotypes of *Plasmodium falciparum in vitro* cultures during gametocyte development

**DOI:** 10.1186/1475-2875-13-468

**Published:** 2014-12-01

**Authors:** Sabrina D Lamour, Ursula Straschil, Jasmina Saric, Michael J Delves

**Affiliations:** Division of Computational and Systems Medicine, Department of Surgery and Cancer, Imperial College London, London, SW7 2AZ UK; Department of Life Sciences, Imperial College London, London, SW7 2AZ UK

**Keywords:** *Plasmodium*, Gametocyte, Gametocytogenesis, Culture, Metabolic, Asexual, Sexual, Maturation, Differentiation, Nutritional

## Abstract

**Background:**

Gametocytes are the *Plasmodium* life stage that is solely responsible for malaria transmission. Despite their important role in perpetuating malaria, gametocyte differentiation and development is poorly understood.

**Methods:**

To shed light on the biochemical changes that occur during asexual and gametocyte development, metabolic characterization of media from *in vitro* intra-erythrocytic *Plasmodium falciparum* cultures was performed throughout gametocyte development by applying ^1^H nuclear magnetic spectroscopy, and using sham erythrocyte cultures as controls. Spectral differences between parasite and sham cultures were assessed via principal component analyses and partial-least squares analyses, and univariate statistical methods.

**Results:**

Clear parasite-associated changes in metabolism were observed throughout the culture period, revealing differences between asexual parasites and gametocyte stages. With culture progression and development of gametocytes, parasitic release of the glycolytic end products lactate, pyruvate, alanine, and glycerol, were found to be dramatically reduced whilst acetate release was greatly increased. Also, uptake of lipid moieties CH_2_, CH_3_, and CH = CH-CH_2_-CH_2_ increased throughout gametocyte development, peaking with maturity.

**Conclusions:**

This study uniquely presents an initial characterization of the metabolic exchange between parasite and culture medium during *in vitro P. falciparum* gametocyte culture. Results suggest that energy metabolism and lipid utilization between the asexual stages and gametocytes is different. This study provides new insights for gametocyte-specific nutritional requirements to aid future optimization and standardization of *in vitro* gametocyte cultivation, and highlights areas of novel gametocyte cell biology that deserve to be studied in greater detail and may yield new targets for transmission-blocking drugs.

**Electronic supplementary material:**

The online version of this article (doi:10.1186/1475-2875-13-468) contains supplementary material, which is available to authorized users.

## Background

*Plasmodium*, the causative agent of malaria was reported to be responsible for 627,000 deaths in 2012 [1]. Disease symptoms are largely caused by progressive cycles of parasite invasion of erythrocytes in the bloodstream through what is known as the asexual cycle. With every 48-hour asexual cycle, ~0.1-0.2% of *Plasmodium falciparum* asexual parasites become committed to an alternative developmental path, forming the sexual stages of the parasite called gametocytes [2]. The trigger for this transformation is not well understood, however it is thought to be upregulated in response to stress signals induced by high parasitaemia in the human host [3] and is regulated by the Api2G transcription factor [4, 5]. Gametocytes are the *Plasmodium* life stage solely responsible for onward transmission to the mosquito and do not participate in disease symptoms [6].

In *P. falciparum*, gametocytes take about ten days to develop to maturity [2]. During this time they undergo distinct morphological changes defined as Stages I-V. Stages I-III are thought to share similar metabolic activity to asexual parasites, e.g., utilizing erythrocyte haemoglobin as a major amino acid source. Furthermore, they also share similar sensitivity to chloroquine treatment which inhibits haem detoxification [7]. As gametocytes mature, they dramatically reduce their activity and become quiescent, waiting for uptake into the mosquito during a blood feed before continuing development with explosive colonization of the mosquito midgut [8]. Likely due to their arrested state, mature Stage V gametocytes are insensitive to most currently used anti-malarials [9–11]. This leads to a situation where an infected individual can be cured of their disease symptoms but still be infectious to mosquitoes, thus able to perpetuate the cycle of infection. The complex and changing cell biology of the gametocyte highlights the utmost importance of better understanding gametocyte development *in vitro,* to best enable the development and implementation of transmission-blocking interventions.

The laboratory study of *P. falciparum* asexual parasites has been greatly facilitated by their ability to be continuously cultured *in vitro* if supplied regularly with fresh erythrocytes to invade, and supported by appropriate medium [12]. Although simplified protocols for gametocyte cultivation exist and external factors have been somewhat optimized, the infectivity of these cultured gametocytes varies wildly both between experiments [13] and externally between research groups, despite using similar protocols [14]. Considering the differences in length of development, cell biology and extracellular environment that asexual parasites and gametocytes experience *in vivo*, it is likely that the metabolic activity and nutritional requirements of asexual parasites and developing gametocytes is different.

Metabolic profiling studies, using spectroscopic methods such as nuclear magnetic resonance (NMR), mass spectrometry or capillary electrophoresis-based methods, provide powerful tools in capturing the relative levels of whole variety of small metabolites simultaneously. Such work has initially been applied to a variety of *in vivo* models, resulting in the discovery of complex metabolic host-parasite interactions and candidate biomarkers associated with infection and/or disease, including several malaria rodent models [15, 16]. More recently, this approach has been applied to *in vitro* infection models to address more targeted metabolic questions on specific infection-induced changes in host cells [17, 18] or responses towards drug treatment [19], and has been increasingly implemented to characterize blood-stage *P. falciparum* cultures [20–23].

Here a standard ^1^H NMR metabolic profiling approach is used to characterize changes in medium over the entire course of *P. falciparum* gametocyte development *in vitro*, from the generation of gametocytes from an initial asexual population until their final maturation stage. The aim of this explorative study is to identify changes in metabolic activity throughout culture and identify key metabolic markers associated with growth and/or differentiation of the various gametocyte stages, which may shed light on optimizing and standardizing *in vitro P. falciparum* gametocyte culture.

## Methods

### Study design

The study was based on two separate experiments. In the first study, eight replicate parasite cultures were grown for 14 days until functionally mature Stage V gametocytes (as evidenced by their ability to undergo gamete formation) predominated. Cultures were induced by seeding *P. falciparum* 3D7 strain asexual parasites at 1% parasitaemia and 4% haematocrit in 10 ml total volume in 25 sq cm flasks under 3% O_2_, 5% CO_2_, 92% N_2_ gas. Culture medium consisted of RPMI medium supplemented with 25 mM HEPES (Life Technologies), 50 mg/l hypoxanthine (Sigma), 2 g/l sodium bicarbonate (Sigma), 10% A+ pooled human serum (Interstate Bloodbank). Total medium replacement was performed every 24 hours whilst maintaining a culture temperature of 37°C. Aliquots of fresh culture medium not exposed to parasites were used as a baseline control (*n* = 8). Each day, during routine daily medium replacement, a 1 ml sample of spent medium from each culture was collected. Samples were then centrifuged at 12,000 × *g* for 5 minutes to pellet any contaminating cell debris and supernatants were transferred to fresh tubes and stored at -80°C until data acquisition.

To control for any potential changes in the baseline metabolism of the uninfected erythrocyte over the 14-day culture period which could confound the analysis of the parasite metabolism, a second sham experiment was performed in which uninfected erythrocytes were cultured identically as for the first experiment. Culture media samples were collected on days 1, 4, 7, 8, and 14 of the study (*n* = 8 per time-point, 40 in total), along with aliquots of the fresh medium, taken at the same time-point as for the control erythrocytes (*n* = 40 in total). Samples were processed identically as described for the first study. In parallel, three gametocyte cultures were initiated, as before, and thin blood smears taken on days 1, 4, 7, 8, and 14. Smears were stained with Giemsa and parasite stages were counted and recorded microscopically. The mean of five fields at x500 magnification was calculated for each slide and parasitaemia expressed as a percentage of total erythrocyte number.

### ^1^H NMR acquisition and processing

Media samples were prepared for NMR analyses based on protocols as described by Beckonert and colleagues [24]. Briefly, 300 μl media samples were mixed 1:1 with NMR phosphate buffer (50% v/v D20 (GOSS Scientific, UK, 0.01% v/v sodium 3-(trimethylsilyl) propionic acid 2,2,3,3-d4 ([TSP)], pH 7.4) and centrifuged at 12,470 × *g* for 5 minutes. Some 550 μl of sample was transferred into 5-mm NMR tubes (Bruker, Germany) and all samples from both studies were run on a Bruker Avance 600 NMR Spectrometer with TXI probe head (Bruker), using XWIN-NMR software (Bruker Biospin, Germany). ^1^H NMR data were acquired by applying a standard one-dimensional (1D) pulse programme for 128 scans (after eight dummy scans), that included water irradiation during the recycle delay, set at 2 seconds (s). The pulse sequence was set to: recycle delay-90°-t-90°-tm-90°-ACQ, whereby 90° pulse length was set to between 16.5 μs, t (short delay) = 2 s, tm (mixing time) = 100 ms and ACQ (acquisition period) = at 2.73 s per scan. Spectral data underwent baseline correction, internal reference (TSP) peak calibration and phasing, using an in-house MATLAB algorithm (version R2012b, Mathworks Inc, USA) and Topspin 3.1 software (Bruker BioSpin, Germany). Water regions and HEPES buffer peaks were removed followed by to automatic spectral alignment and probabilistic quotient normalization [25] in MATLAB.

### Statistical data analysis

Metabolic spectra of raw data for each study, as well as data of the two combined studies, were analysed via principal component analysis (PCA), an unsupervised pattern-recognition model used to show an unbiased overview of overall spectral variability of the dataset [26]. Using PCA, the total spectral information of each individual sample becomes down-projected as a single coordinate on the PCA scores plot. Metabolic information on those components responsible for driving the groupings/separations in the scores plot can be derived from the corresponding PCA loadings plot. Data were then subjected to partial-least squares discriminatory analyses (PLS-DA), a supervised model that incorporates class assignment, in order to distinguish any subtle metabolic differences between groups [26]. Both PCA and PLS-DA were performed with SIMCA P+ software (Version 13.0, Umetrics, Sweden).

Specific spectral differences between media from parasite samples and fresh media were further analysed using orthogonal PLS-DA [26], to assess metabolites that were consumed/released into the media during parasite culture. Significantly differential metabolites were selected based on their peak having a Pearson-product moment correlation coefficient (R) that surpassed 0.5, corresponding to the approximate minimum critical values for p < 0.05, and ROC sensitivity/specificity score of at least 0.8. Metabolites were assigned using NMR suite profiler 7.0 software (Chenomx, USA) and referring to inhouse NMR databases and RPMI media formulation sheets from supplier.

Levels of differential metabolites were calculated by measuring metabolite peak integrals across parasite cultures, uninfected erythrocytes and fresh media samples. To account for media batch differences between the two studies, mean average integrals of fresh culture media from each study were subtracted from each metabolite integral of their respective cultures, for each time-point. Media-adjusted metabolite integrals from parasite and erythrocyte cultures were compared for each metabolite, at each matching time-point, by applying non-parametric Mann-Whitney U-test, followed by Benjamini-Hochberg false discovery rate correction [27], to determine significant changes in metabolism linked with *P. falciparum* infection. Parasite-associated changes in metabolites (i.e., media-adjusted levels from gametocytes minus media-adjusted levels from time-matched control erythrocytes) were subsequently divided by total parasitaemia percentages for each corresponding time-point.

## Results

### Parasite developmental changes are reflected by alterations in metabolism

**Figure 1:**
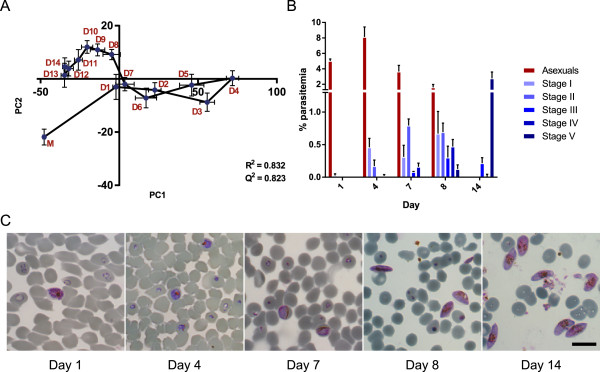
**The relationship between metabolic and developmental changes during** ***in vitro*** **parasite culture. (A)** Metabolic time-trajectory showing the first two components (PC1 and PC2) of a PCA scores plot on fresh culture medium (M) and parasite-conditioned medium from intra-erythrocytic *P. falciparum* cultures, throughout the 14-day culture period (D1-D14). *n* = 8 samples for each time-point. Coordinates and error bars represent mean averages for each time point and standard error of the mean (SEM), respectively. R^2^ and Q^2^ represent the values of the degrees of model fit and predictive values of the PCA model for components 1 and 2, respectively. **(B)** Parasite populations at culture days representing major shifts in trajectory (D1, D4, D7, D8, D14) were assessed by manual counting of Giemsa-stained culture smears (n = 3 cultures, showing mean ± SEM). Days 1-4 were typified by an increasing asexual population; Days 4-7 showed a decline in asexuals and emergence of early gametocytes; Days 7-14 showed gametocyte maturation; by Day 14, the culture consisted mainly of mature Stage V gametocytes. **(C)** Representative microscope images showing parasite culture composition at the culture days assessed. Scale bar = 10 μm.

Media from parasite cultures were collected every 24 hours throughout the 14-day culture period until gametocyte maturation, and metabolically phenotyped using ^1^H NMR spectroscopy. PCA analysis showed a clear separation between the cultures and fresh medium (Figure 1A), which according to the loadings was driven primarily by higher glucose levels in the fresh medium (Additional file 1). Furthermore, striking changes were also observed between time-points within the cultures. From Day 1 the model showed a strong shift in metabolic phenotype along the first component, relative to fresh medium, until Day 4 of culture. Parasitaemia counts revealed that Day 4 corresponded to the peak in asexual population (Figure 1B and C) as well as initial appearance of ‘early’ gametocytes (Stage I-II). As the asexual parasite populations reduced from 8.1% (± 1.3% standard error, SEM) at Day 4 to 3.6% (± 0.8% SEM) by Day 7, early gametocyte numbers continued to increase. During this period, the overall culture metabolism reverted back, similar to the initial phenotype (Figure 1A).

Interestingly, a different metabolic shift was observed from Day 8 onwards, correlating with continued gametocyte generation and the emergence of intermediate stage (III-IV) gametocytes (Figure 1B and C). Metabolic profiles from Days 12 to 14 largely clustered together, with little difference to fresh medium along the first component, thus confirming that the final stages of gametocyte maturation share a similar metabolic activity. By Day 14, asexual parasites were completely absent from the culture and most gametocytes were mature (Stage V; see Figure 1C), resulting in a final mature gametocytaemia of 2.8% (± 0.8% SEM). Corresponding PCA loadings revealed that release of lactate and pyruvate was closely linked with the early shift during Days 1-4, whilst acetate production was associated with the later stages of development to maturity (Additional file 1).

### *Plasmodium falciparum*infection associated with increased intra-erthrocytic metabolic activity

**Figure 2:**
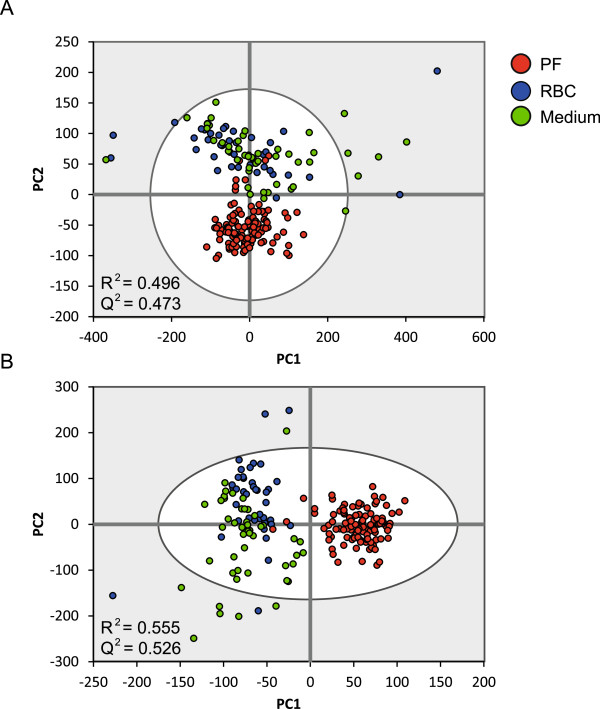
**Variance of dataset dominated by infection status.** PCA model **(A)** and PLS-DA model **(B)** score plots of ^1^H NMR spectra across all time-points from both studies. *Plasmodium falciparum* parasite cultures (PF) in red (*n* = 112), uninfected erythrocyte cultures (RBC) in blue (*n* = 40) and fresh media samples are coloured green (*n* = 48). Cumulative values of model fit (R^2^) and model prediction (Q^2^) for the first two components are denoted on both plots.

To adjust for the potential basal metabolic changes of uninfected erythrocytes, which comprise the large bulk of the parasite cultures, sham cultures of uninfected erythrocytes were generated using identical conditions. Having established that major metabolic changes occur at Days 1, 4, 7, 8, and 14 of parasite culture (Figure 1), medium from these time-points was selected for further analysis. Spectra of parasite cultures, uninfected erythrocytes and fresh culture medium from all assessed time-points were analysed using PCA. Gametocyte cultures clearly separated from the other two groups, whilst control erythrocytes and fresh medium broadly co-localized (Figure 2A). This suggests that a relatively low background metabolic activity was exerted by uninfected erythrocytes, with only a minor contribution to the total observed metabolic activity in the parasite cultures. Further investigation with PLS-DA showed an enhanced separation of parasite cultures from the other samples but also revealed partial separation between the uninfected cultures and fresh medium (Figure 2B). Additional analyses on fresh medium samples alone via PCA revealed subtle batch differences, particularly between the two studies (see Additional file 2) and was thus accounted for in subsequent comparative analyses between the gametocyte and control erythrocyte cultures (see Methods).

### Differential metabolic markers are associated with asexual growth and gametocyte maturation

**Figure 3:**
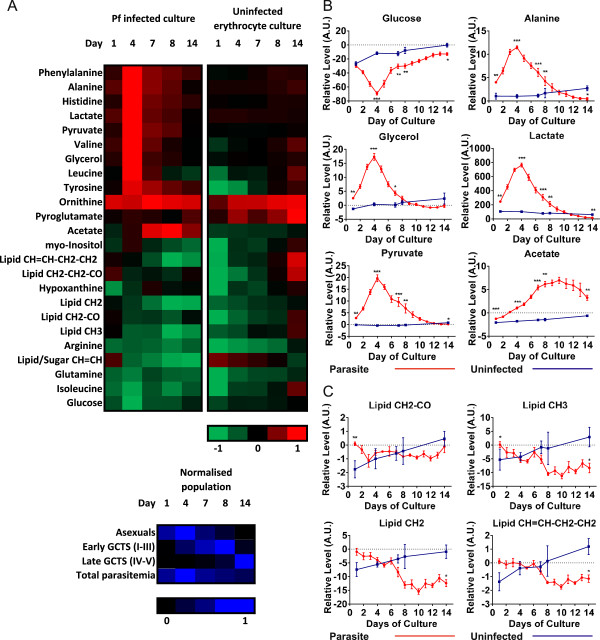
**Metabolites associated with different developmental stages of** ***Plasmodium falciparum*** **within** ***in vitro*** **gametocyte cultures. (A)** Medium metabolite levels for *P. falciparum* (Pf) cultures and uninfected erythrocyte cultures sampled during culture progression. Red indicates metabolite consumed from medium, green indicates released into medium. For comparison, culture parasite stage composition for the different time points is indicated in blue; GCT, gametocytes. **(B)** Culture metabolite trajectories for glucose and reported *Plasmodium* glycolytic endpoints. **(C)** Culture metabolite trajectories for selected lipid moieties. Graphs show means ± SEM as error bars. *P < 0.05; **P < 0.01; ***P < 0.001 Mann-Whitney U test.

Multivariate discriminatory analysis (using orthogonal PLS-DA) was performed between fresh medium and spent medium of parasite cultures, to identify metabolites that were consumed and/or released during culture. In order to separate parasite-associated changes in metabolism from that of uninfected erythrocytes, integrals of these were calculated and differences between time-point-matched parasite and control erythrocyte cultures were investigated (Figure 3 and Table 1).

**Table 1:** **Metabolites significantly altered by** ***Plasmodium falciparum*** **infection throughout gametocyte culture period**

	Metabolite	*p* -values				
		Day 1	Day 4	Day 7	Day 8	Day 14
Metabolites released into medium	Acetate	0.0046	0.0005	0.0008	0.0024	0.0024
	Alanine	0.0012	0.0005	0.0008	NS	0.0188 (-)
	Glycerol	0.0012	0.0005	0.0153	NS	NS
	Histidine	0.0129	0.0005	0.0008	0.0036	NS
	Lactate	0.0012	0.0005	0.0008	0.0024	0.0024 (-)
	Ornithine	0.0129	NS	NS	NS	NS
	Phenylalanine	0.0014	0.0005	0.0008	0.0188	NS
	Pyroglutamate	NS	NS	NS	0.0444 (-)	0.0210 (-)
	Pyruvate	0.0012	0.0005	0.0008	0.0024	0.0250 (-)
	Tyrosine	0.0046	0.0007	0.0090	NS	NS
	Valine	0.0033	0.0005	0.0153	NS	NS
Metabolites consumed from medium	Arginine	0.0166 (-)	NS	NS	NS	NS
	Glucose	NS	0.0005	0.0038	0.0091	0.0152
	Glutamine	0.0033 (-)	NS	NS	NS	NS
	Hypoxanthine	NS	NS	0.0125 (-)	NS	NS
	*myo*-Inositol	NS	NS	NS	NS	0.0210 (-)
	Isoleucine	NS	0.0012	NS	NS	0.0250
	Lipid/Sugar CH = CH	NS	NS	NS	NS	NS
	Lipid CH = CH-CH2-CH2	NS	NS	NS	NS	0.0414
	Lipid CH2	NS	NS	NS	NS	0.0414
	Lipid CH2-CH2-CO	0.0222 (-)	NS	NS	NS	NS
	Lipid CH2-CO	0.0033 (-)	NS	NS	NS	NS
	Lipid CH3	0.0397 (-)	NS	NS	NS	0.0180
Variable consumption/release	Leucine	0.0065 (-)	0.0005	0.0296	NS	0.0188 (-)

Statistical differences between metabolite levels of medium from parasite cultures and uninfected erythrocytes, assessed for each time-point as measured via Mann-Whitney U-test. *P*-values indicate significantly higher metabolite release/consumption by parasite cultures, unless marked with (-), where release/consumption was lower than erythrocytes. NS, no significant difference.

One of the most notable changes appears to be linked to glucose utilization. Glucose consumption was significantly higher in parasite cultures from Days 4-14, peaking at Day 4 (Figure 3B), consistent with maximal parasitaemia (Figure 3A). Following normalization to parasite counts, relative stable glucose consumption from Days 4-14 was observed (Additional file 3), suggesting that asexuals and gametocytes of all stages of development have similar glucose requirements. Known metabolites of glucose utilization in *Plasmodium* asexual parasites – alanine, glycerol, lactate, and pyruvate [28] were all maximally released by the parasites at Day 4, before declining to the basal erythrocyte level (or below) by Day 14 (Figure 3B). When total culture parasitaemia was accounted for, all still showed a maximal peak of release at Day 4, with little to no release by Day 14 (Additional file 3). In contrast to other glycolytic end-products, acetate, which has been linked to glucose utilization in gametocytes [22] was released by the parasites only at very low levels early in culture, was maximal later in culture, before slightly declining again at Day 14 (Figure 3B). This trajectory was mirrored even after normalizing for parasitaemia (Additional file 3).

The amino acids histidine, phenylalanine, tyrosine, valine, and leucine were all maximally released into the medium at Day 4 before sharply declining to basal erythrocyte levels by Day 14 (Figure 3A, Additional file 3). Accounting for total parasitaemia, peak histidine and phenylalanine release occurred at Day 4 and gradually declined. Leucine, tyrosine and valine release was relatively stable at Days 1-7 before sharply dropping to basal erythrocyte levels or below at Days 8-14 (Additional file 3). In contrast, isoleucine, an amino acid known to be essential for parasite growth [29], was consumed at all time-points, with statistical significance observed on Days 4 and 14. When adjusted for culture parasitaemia, isoleucine consumption was maximal at Day 14 (Figure 3A, Additional file 3).

Generally, identified lipid moieties fell into two categories: those that showed little or no difference from basal erythrocyte consumption levels, such as CH_2_-CO, CH_2_-CH_2_-CO and CH = CH; and those that increased in consumption with gametocyte maturation from Day 8 onwards (Figure 3A and C, Additional file 3). The latter category included lipid methyl groups (CH_3_), methylene groups (CH_2_,) and CH = CH-CH_2_-CH_2_ fractions (Figure 3C), whereby the increase in consumption was significant by Day 14 of culture (Table 1).

## Discussion

The generation of *P. falciparum* gametocytes *in vitro* involves the initial rapid growth of intra-erythrocytic asexual parasites, sexual commitment, which is then followed by gradual maturation [3]. This study surveys the extracellular metabolic changes throughout gametocyte culture development through analysis of spent culture medium *via*^1^H NMR spectroscopy. Using this approach has identified clear differences in metabolic activity between asexual and gametocyte growth which is broadly consistent with other studies [22, 28].

The most distinct difference observed appeared to be associated with parasite glucose utilization. Given the abundance of glucose in serum, the asexual parasite relies entirely on glycolysis for energy production [30]. Four previously confirmed glycolytic fermentation end-products (lactate, pyruvate, alanine, and glycerol [28]) were identified as released into the culture medium coinciding with the peak asexual population. Whilst culture glucose consumption continued throughout gametocyte maturation, as the asexual population declined the release of these metabolites decreased and was replaced by the production of acetate. Acetate is a common glycolytic end-product of other eukaryotic parasites [31] and has been recently linked to Stage III gametocyte glucose metabolism [22]. Whilst not proven, this shift, linked to *Plasmodium* sexual development, is hypothesized to be a result of increased acetyl-CoA synthesis in the gametocyte mitochondrion or apicoplast [22]. Alternatively, recent work has provided evidence that acetyl-CoA synthetase and (indirectly) histone deacetylation may also contribute to the intracellular acetate pool in asexual parasites [32], and perhaps this is upregulated during gametocyte development. Additionally, acetate may be released during parasite GPI anchor biosynthesis [33].

Indicative of an abundance of amino acids available from haemoglobin digestion, asexual growth appears to be generally linked to the release of amino acids into the culture medium rather than their consumption. In support of this, a previous metabolomic study that focused on asexual intra-erythrocytic development using mass spectroscopy found similarly that most amino acids are released into the culture medium during the asexual cycle [21]. Presented here, the release of the majority of amino acids was at Day 4 when peak asexual parasitaemia is reached and gametocytes first arise (Figure 3A). Given that asexual parasitaemia subsequently begins to sharply decline, it cannot be ruled out that the bulk metabolite release at this time-point may simply be caused by cell lysis through apoptosis/necrosis rather than productive metabolism. In either case, elevated levels of these metabolites could signal to induce an upregulation of commitment to gametocyte development or alternatively could be toxic and detrimental to *in vitro* gametocyte culture (as has recently shown for lactate which retards asexual growth [34]).

Medium depletion of several lipid moieties was found to be associated with gametocyte maturation (Figure 3C). *Plasmodium* has the ability to generate some fatty acids *de novo* in the apicoplast [35] but most are scavenged from host serum and modified or utilized directly [36]. Transcriptomic analysis indicates that five of the six members of the type II fatty acid pathway are upregulated during gametocyte development [37]. Also, it has recently been shown that the lipid transporter gABC2 plays a role in the accumulation of neutral lipids within the parasite and is expressed predominantly in maturing female gametocytes [38]. Both of these support the assertion that lipid metabolism is important for gametocyte development and is different from asexual parasites.

One of the most remarkable morphological changes that occurs during gametocyte development is extensive mitochondrial elongation, branching and the formation of multiple mitochondrial cristate structures [39, 40]. Supported by the shift towards glycolytic acetate production, which suggests fundamental changes in the way parasites generate energy during gametocyte development, it is tempting to speculate that this mitochondrial transformation burdens the developing gametocyte with increased requirements for membrane production and concomitantly increases their demand for exogenous lipids. As such, parasite energy production or lipid metabolism may be vulnerable targets in the generally drug-insensitive stage V mature gametocyte and could suggests avenues for drug discovery.

## Conclusions

Very little is known about gametocyte development at the biochemical level. This preliminary study identifies and links the developmental flux of key metabolites to parasites in *in vitro* gametocyte cultures. It is clear that the metabolism of gametocytes is divergent from asexual parasites. Despite these differences, the composition of conventional parasite culture medium remains constant throughout the culture period. Clearly viable gametocytes develop under these conditions, as evidenced by their onward infectivity to mosquitoes, however cultured gametocytes are less infective than those that have developed in the human host [41] suggesting suboptimal growth conditions. With this in mind, it is hoped that data presented will help focus attention towards the development of gametocyte-specific medium formulations.

To this effect, supporting evidence is provided that gametocytes utilize acetate as an end-product for glucose metabolism and that gametocyte lipid requirements are divergent from asexual parasites. Consequently, it is suggested that further investigations of acetate and fatty acid metabolism in particular would significantly benefit current understanding on gametocyte developmental cell biology. Additional ^13^C-labelling metabolic studies and/or proteomic investigations may also help elucidate role of acetate metabolism, whilst other targeted metabolic profiling techniques, such as reverse phase liquid chromatography-mass spectrometry (LC-MS), could provide a more comprehensive characterization and identification of the different lipid species in question. Together, such studies would generate a better understanding of gametocyte development and will greatly aid both optimization of expensive and variable gametocyte culture for future laboratory investigations, helping to shed light on potential new targets for transmission-blocking drugs.

## Electronic supplementary material

Additional file 1: **Key metabolites responsible for phenotypic shifts observed within PCA time** **-** **trajectory of parasite cultures.** PCA loadings plot showing spectral peaks responsible for driving the variability of parasite conditioned media in the first two components (p[1] and p[2]), observed in the corresponding scores plot shown in Figure 1A. Metabolites of the peaks the most strongly associated with the observed shifts in metabolic phenotypes have been labelled on the plot. Abbreviations: BCAA, branched chain amino acid peaks (valine, leucine, isoleucine). (PDF 129 KB)

Additional file 2: **Variability in media composition between the two studies.** PCA scores plot showing components 1 *vs* 2 **(A)** and 3 vs 4 **(B)** of fresh media samples from the two studies. Partial separation of Study 1 Day 0 media samples from the remaining samples apparent from components 3 and 4. R2 and Q2 represent the values of the degrees of model fit and predictive values of the PCA model, respectively, for each of the four components. (PDF 119 KB)

Additional file 3: **Trajectories of highlighted metabolites adjusted for parasitaemia.** Graphs showing culture metabolite levels over time for media from parasite-infected cultures (red) and control uninfected erythrocyte cultures (blue) as determined by multivariate discriminatory analysis (orthogonal PLAS-DA). *n*=8, with standard error of the mean as error bars. Control culture metabolite levels were subtracted from parasite-infected cultures and then normalised to the total parasitemia counts derived from Figure 1B (green). (PDF 165 KB)

## References

[1] WHO (2013). World Malaria Report 2013.

[2] Sinden RE (1983). Sexual development of malarial parasites. Adv Parasitol.

[3] Baker DA (2010). Malaria gametocytogenesis. Mol Biochem Parasitol.

[4] Kafsack BFC, Rovira-Graells N, Clark TG, Bancells C, Crowley VM, Campino SG, Williams AE, Drought LG, Kwiatkowski DP, Baker DA, Cortés A, Llinás M (2014). A transcriptional switch underlies commitment to sexual development in malaria parasites. Nature.

[5] Sinha A, Hughes KR, Modrzynska KK, Otto TD, Pfander C, Dickens NJ, Religa AA, Bushell E, Graham AL, Cameron R, Kafsack BFC, Williams AE, Llinás M, Berriman M, Billker O, Waters AP (2014). A cascade of DNA-binding proteins for sexual commitment and development in Plasmodium. Nature.

[6] Dixon MWA, Thompson J, Gardiner DL, Trenholme KR (2008). Sex in Plasmodium: a sign of commitment. Trends Parasitol.

[7] Sinden RE (1982). Gametocytogenesis of *Plasmodium falciparum* in vitro: ultrastructural observations on the lethal action of chloroquine. Ann Trop Med Parasitol.

[8] Sinden RE, Smalley ME (1979). Gametocytogenesis of *Plasmodium falciparum* in vitro: the cell-cycle. Parasitology.

[9] Smalley ME (1977). *Plasmodium falciparum* gametocytes: The effect of chloroquine on their development. Trans R Soc Trop Med Hyg.

[10] Chutmongkonkul M, Maier WA, Seitz HM (1992). A new model for testing gametocytocidal effects of some antimalarial drugs on *Plasmodium falciparum* in vitro. Ann Trop Med Parasitol.

[11] Kumar N, Zheng H (1990). Stage-specific gametocytocidal effect in vitro of the antimalaria drug qinghaosu on *Plasmodium falciparum*. Parasitol Res.

[12] Trager W, Jensen JB (1976). Human malaria parasites in continuous culture. Science.

[13] Ponnudurai T, Lensen AH, Van Gemert GJ, Bensink MP, Bolmer M, Meuwissen JH (1989). Infectivity of cultured *Plasmodium falciparum* gametocytes to mosquitoes. Parasitology.

[14] Churcher TS, Blagborough AM, Delves M, Ramakrishnan C, Kapulu MC, Williams AR, Biswas S, Da DF, Cohuet A, Sinden RE (2012). Measuring the blockade of malaria transmission - an analysis of the standard membrane feeding assay. Int J Parasitol.

[15] Li JV, Wang Y, Saric J, Nicholson JK, Dirnhofer S, Singer BH, Tanner M, Wittlin S, Holmes E, Utzinger J (2008). Global metabolic responses of NMRI mice to an experimental *Plasmodium berghei* infection. J Proteome Res.

[16] Ghosh S, Sengupta A, Sharma S, Sonawat HM (2011). Multivariate modelling with (1)H NMR of pleural effusion in murine cerebral malaria. Malar J.

[17] Lamour SD, Choi B-S, Keun HC, Müller I, Saric J (2012). Metabolic characterization of *Leishmania major i*nfection in activated and nonactivated macrophages. J Proteome Res.

[18] Birungi G, Chen SM, Loy BP, Ng ML, Li SFY (2010). Metabolomics approach for investigation of effects of dengue virus infection using the EA.hy926 cell line. J Proteome Res.

[19] Vincent IM, Creek DJ, Burgess K, Woods DJ, Burchmore RJS, Barrett MP (2012). Untargeted metabolomics reveals a lack of synergy between nifurtimox and eflornithine against *Trypanosoma brucei*. PLoS Negl Trop Dis.

[20] Teng R, Junankar PR, Bubb WA, Rae C, Mercier P, Kirk K (2009). Metabolite profiling of the intraerythrocytic malaria parasite *Plasmodium falciparum* by (1)H NMR spectroscopy. NMR Biomed.

[21] Olszewski KL, Morrisey JM, Wilinski D, Burns JM, Vaidya AB, Rabinowitz JD, Llinás M (2009). Host-parasite interactions revealed by *Plasmodium falciparum* metabolomics. Cell Host Microbe.

[22] MacRae JI, Dixon MW, Dearnley MK, Chua HH, Chambers JM, Kenny S, Bottova I, Tilley L, McConville MJ (2013). Mitochondrial metabolism of sexual and asexual blood stages of the malaria parasite *Plasmodium falciparum*. BMC Biol.

[23] Sana TR, Gordon DB, Fischer SM, Tichy SE, Kitagawa N, Lai C, Gosnell WL, Chang SP (2013). Global mass spectrometry based metabolomics profiling of erythrocytes infected with *Plasmodium falciparum*. cbvPLoS One.

[24] Beckonert O, Keun HC, Ebbels TMD, Bundy J, Holmes E, Lindon JC, Nicholson JK (2007). Metabolic profiling, metabolomic and metabonomic procedures for NMR spectroscopy of urine, plasma, serum and tissue extracts. Nat Protoc.

[25] Dieterle F, Ross A, Schlotterbeck G, Senn H (2006). Probabilistic quotient normalization as robust method to account for dilution of complex biological mixtures. Application in 1H NMR metabonomics. Anal Chem.

[26] Trygg J, Holmes E, Lundstedt T (2007). Chemometrics in metabonomics. J Proteome Res.

[27] Benjamini Y, Hochberg Y (1995). Controlling the false discovery rate: a practical and powerful approach to multiple testing. J R Stat Soc Ser B Methodol.

[28] Lian L-Y, Al-Helal M, Roslaini AM, Fisher N, Bray PG, Ward SA, Biagini GA (2009). Glycerol: An unexpected major metabolite of energy metabolism by the human malaria parasite. Malar J.

[29] Istvan ES, Dharia NV, Bopp SE, Gluzman I, Winzeler EA, Goldberg DE (2011). Validation of isoleucine utilization targets in *Plasmodium falciparum*. Proc Natl Acad Sci U S A.

[30] van Schalkwyk DA, Priebe W, Saliba KJ (2008). The inhibitory effect of 2-halo derivatives of d-glucose on glycolysis and on the proliferation of the human malaria parasite *Plasmodium falciparum*. J Pharmacol Exp Ther.

[31] Tielens AGM, van Grinsven KWA, Henze K, van Hellemond JJ, Martin W (2010). Acetate formation in the energy metabolism of parasitic helminths and protists. Int J Parasitol.

[32] Cobbold SA, Vaughan AM, Lewis IA, Painter HJ, Camargo N, Perlman DH, Fishbaugher M, Healer J, Cowman AF, Kappe SHI, Llinás M (2013). Kinetic flux profiling elucidates two independent acetyl-CoA biosynthetic pathways in *Plasmodium falciparum*. J Biol Chem.

[33] *PF3D7_0624700: (N-acetylglucosaminylphosphatidylinositol deacetylase)*. http://plasmodb.org/plasmo/showRecord.do?name=GeneRecordClasses.GeneRecordClass&source_id=PF3D7_0624700&project_id=PlasmoDB

[34] Hikosaka K, Hirai M, Komatsuya K, Ono Y, Kita K (2014). Lactate retards the development of erythrocytic stages of the human malaria parasite *Plasmodium falciparum*. Parasitol Int.

[35] Lim L, McFadden GI (2010). The evolution, metabolism and functions of the apicoplast. Philos Trans R Soc Lond B Biol Sci.

[36] Mi-Ichi F, Kita K, Mitamura T (2006). Intraerythrocytic *Plasmodium falciparum* utilize a broad range of serum-derived fatty acids with limited modification for their growth. Parasitology.

[37] Young JA, Fivelman QL, Blair PL, de la Vega P, Le Roch KG, Zhou Y, Carucci DJ, Baker DA, Winzeler EA (2005). The *Plasmodium falciparum* sexual development transcriptome: A microarray analysis using ontology-based pattern identification. Mol Biochem Parasitol.

[38] Tran PN, Brown SHJ, Mitchell TW, Matuschewski K, McMillan PJ, Kirk K, Dixon MWA, Maier AG (2014). A female gametocyte-specific ABC transporter plays a role in lipid metabolism in the malaria parasite. Nat Commun.

[39] Krungkrai J (2004). The multiple roles of the mitochondrion of the malarial parasite. Parasitology.

[40] Okamoto N, Spurck TP, Goodman CD, McFadden GI (2009). Apicoplast and mitochondrion in gametocytogenesis of *Plasmodium falciparum*. Eukaryot Cell.

[41] Schneider P, Bousema JT, Gouagna LC, Otieno S, van de Vegte-Bolmer M, Omar SA, Sauerwein RW (2007). Submicroscopic *Plasmodium falciparum* gametocyte densities frequently result in mosquito infection. Am J Trop Med Hyg.

