# Supplementary material for: Changes in metabolic phenotypes of Plasmodium falciparum in vitro cultures during gametocyte development
Source: Malar J. 2014 Dec 1;13:468. doi: 10.1186/1475-2875-13-468 (PMC4289216; doi:10.1186/1475-2875-13-468)

Additional file 1 – Key metabolites responsible for phenotypic shifts observed within PCA time-trajectory of parasite cultures

PCA loadings plot showing spectral peaks responsible for driving the variability of parasite-conditioned media in the first two components (p[1] and p[2]), observed in the corresponding scores plot shown in Figure 1A. Metabolites of the peaks the most strongly associated with the observed shifts in metabolic phenotypes have been labelled on the plot. Abbreviations: BCAA, branched-chain amino acid peaks (valine, leucine, isoleucine).

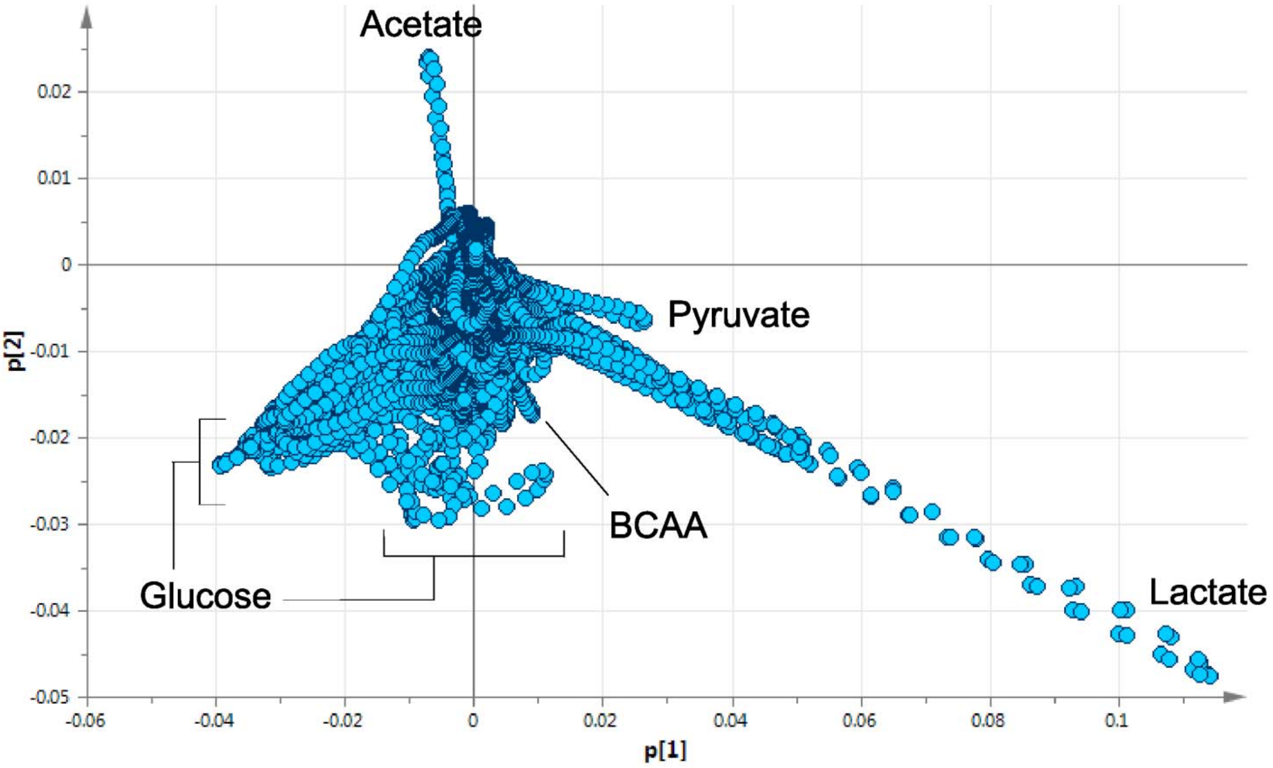

Supplement: Supplementary file 1 — Additional file 1: Key metabolites responsible for phenotypic shifts observed within PCA time - trajectory of parasite cultures. PCA loadings plot showing spectral peaks responsible for driving the variability of parasite conditioned media in the first two components (p[1] and p[2]), observed in the corresponding scores plot shown in Figure 1A. Metabolites of the peaks the most strongly associated with the observed shifts in metabolic phenotypes have been labelled on the plot. Abbreviations: BCAA, branched chain amino acid peaks (valine, leucine, isoleucine). (PDF 129 KB) [file 12936_2014_3652_MOESM1_ESM.pdf]
