# Supplementary material for: Changes in metabolic phenotypes of Plasmodium falciparum in vitro cultures during gametocyte development
Source: Malar J. 2014 Dec 1;13:468. doi: 10.1186/1475-2875-13-468 (PMC4289216; doi:10.1186/1475-2875-13-468)

Additional file 2 – Variability in media composition between the two studies

PCA scores plot showing components 1 *vs* 2 (A) and 3 vs 4 (B) of fresh media samples from the two studies. Partial separation of Study 1 Day 0 media samples from the remaining samples apparent from components 3 and 4. R^2 and Q^2 represent the values of the degrees of model fit and predictive values of the PCA model, respectively, for each of the four components.

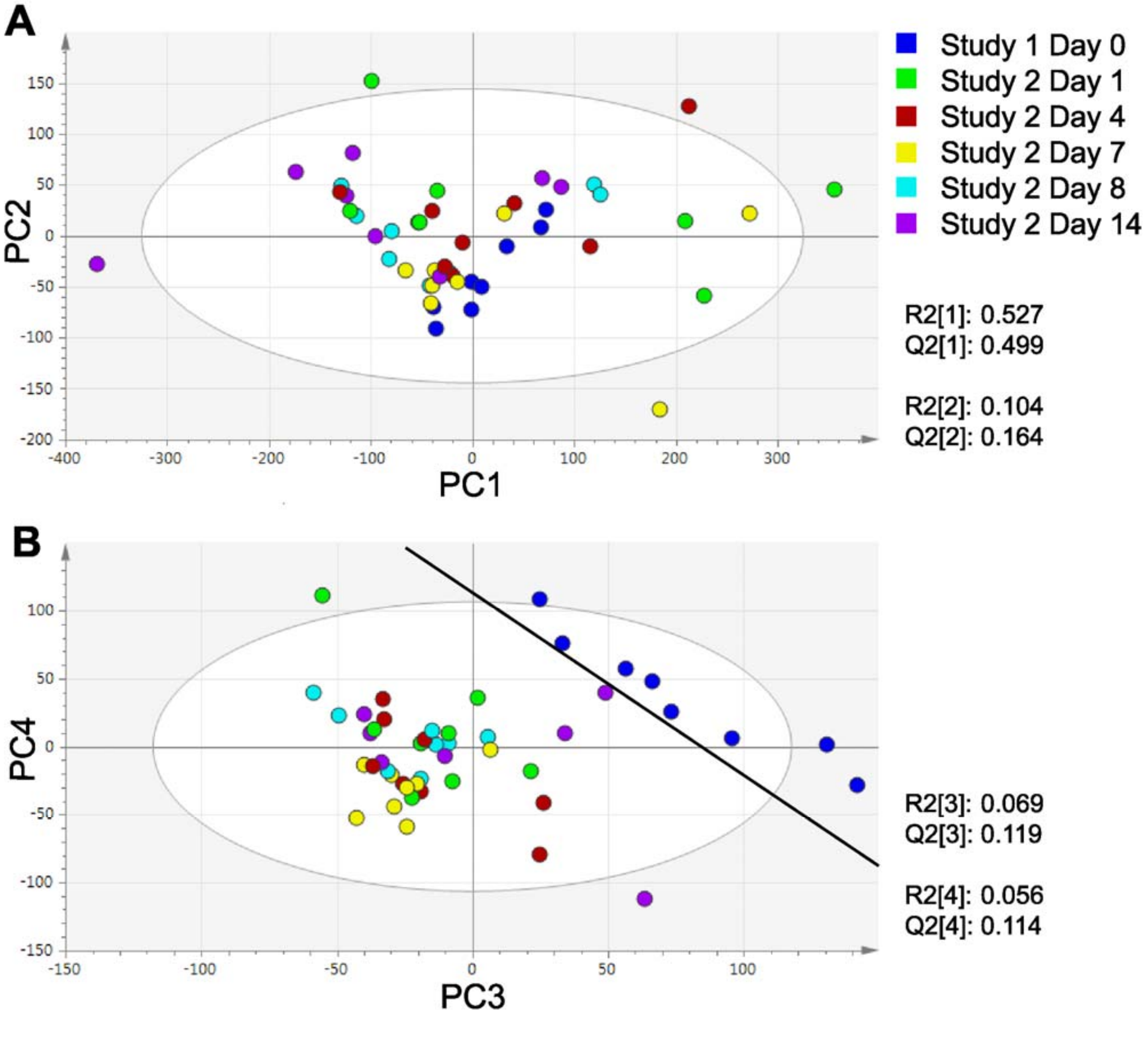

Supplement: Supplementary file 2 — Additional file 2: Variability in media composition between the two studies. PCA scores plot showing components 1 vs 2 (A) and 3 vs 4 (B) of fresh media samples from the two studies. Partial separation of Study 1 Day 0 media samples from the remaining samples apparent from components 3 and 4. R2 and Q2 represent the values of the degrees of model fit and predictive values of the PCA model, respectively, for each of the four components. (PDF 119 KB) [file 12936_2014_3652_MOESM2_ESM.pdf]
