# Supplementary material for: Changes in metabolic phenotypes of Plasmodium falciparum in vitro cultures during gametocyte development
Source: Malar J. 2014 Dec 1;13:468. doi: 10.1186/1475-2875-13-468 (PMC4289216; doi:10.1186/1475-2875-13-468)
Supplement: Supplementary file 3 — Additional file 3: Trajectories of highlighted metabolites adjusted for parasitaemia. Graphs showing culture metabolite levels over time for media from parasite-infected cultures (red) and control uninfected erythrocyte cultures (blue) as determined by multivariate discriminatory analysis (orthogonal PLAS-DA). n=8, with standard error of the mean as error bars. Control culture metabolite levels were subtracted from parasite-infected cultures and then normalised to the total parasitemia counts derived from Figure 1B (green). (PDF 165 KB) [file 12936_2014_3652_MOESM3_ESM.pdf]

Additional file 3 – Trajectories of highlighted metabolites adjusted for parasitaemia

Graphs showing culture metabolite levels over time for media from parasite-infected cultures (red) and control uninfected erythrocyte cultures (blue) as determined by multivariate discriminatory analysis (orthogonal PLAS-DA). *n*=8, with standard error of the mean as error bars. Control culture metabolite levels were subtracted from parasite-infected cultures and then normalised to the total parasitemia counts derived from Figure 1B (green).

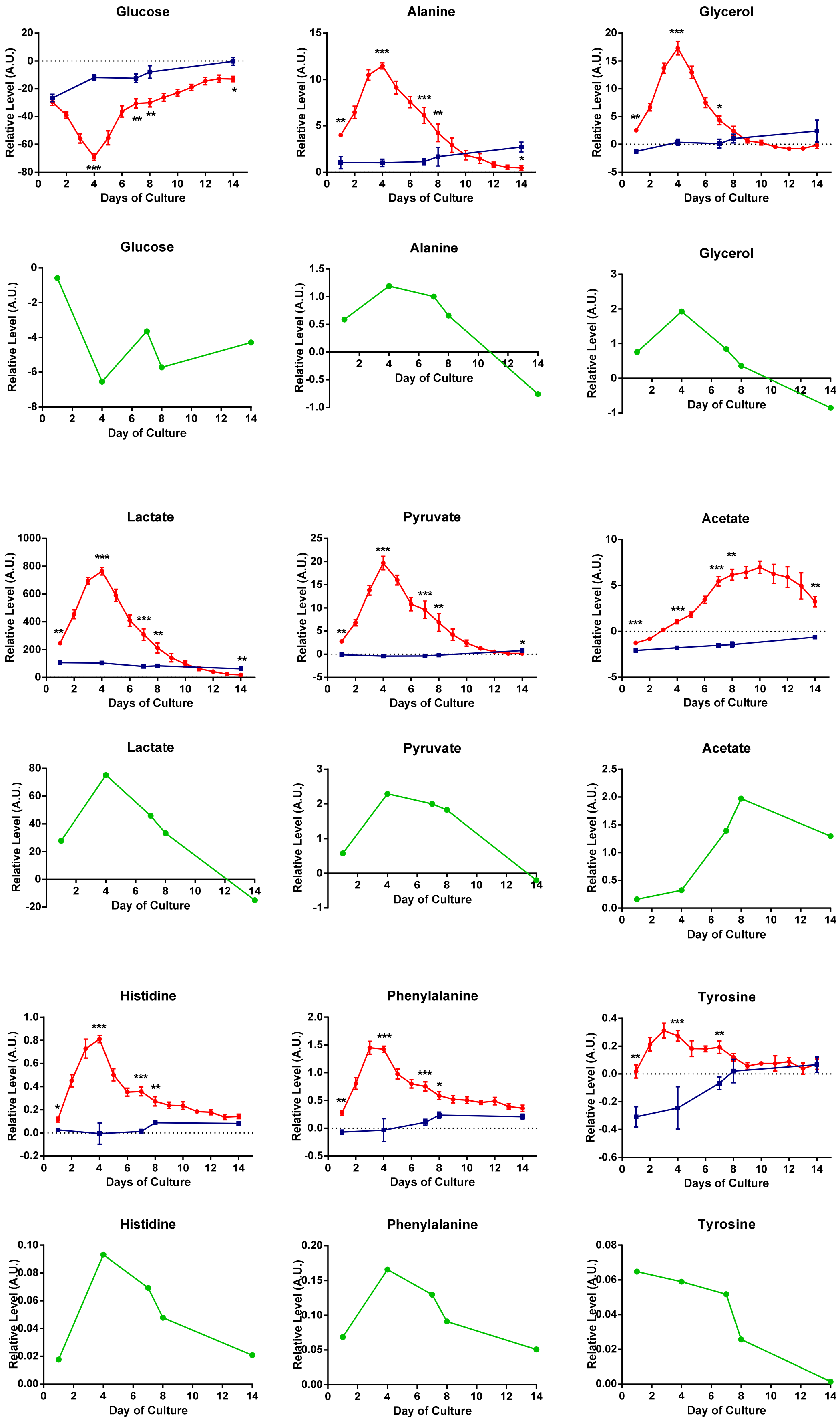
Glucose
Alanine
Glycerol
Relative Level (A.U.)
Days of Culture
Day of Culture
Lactate
Pyruvate
Acetate
Histidine
Phenylalanine
Tyrosine

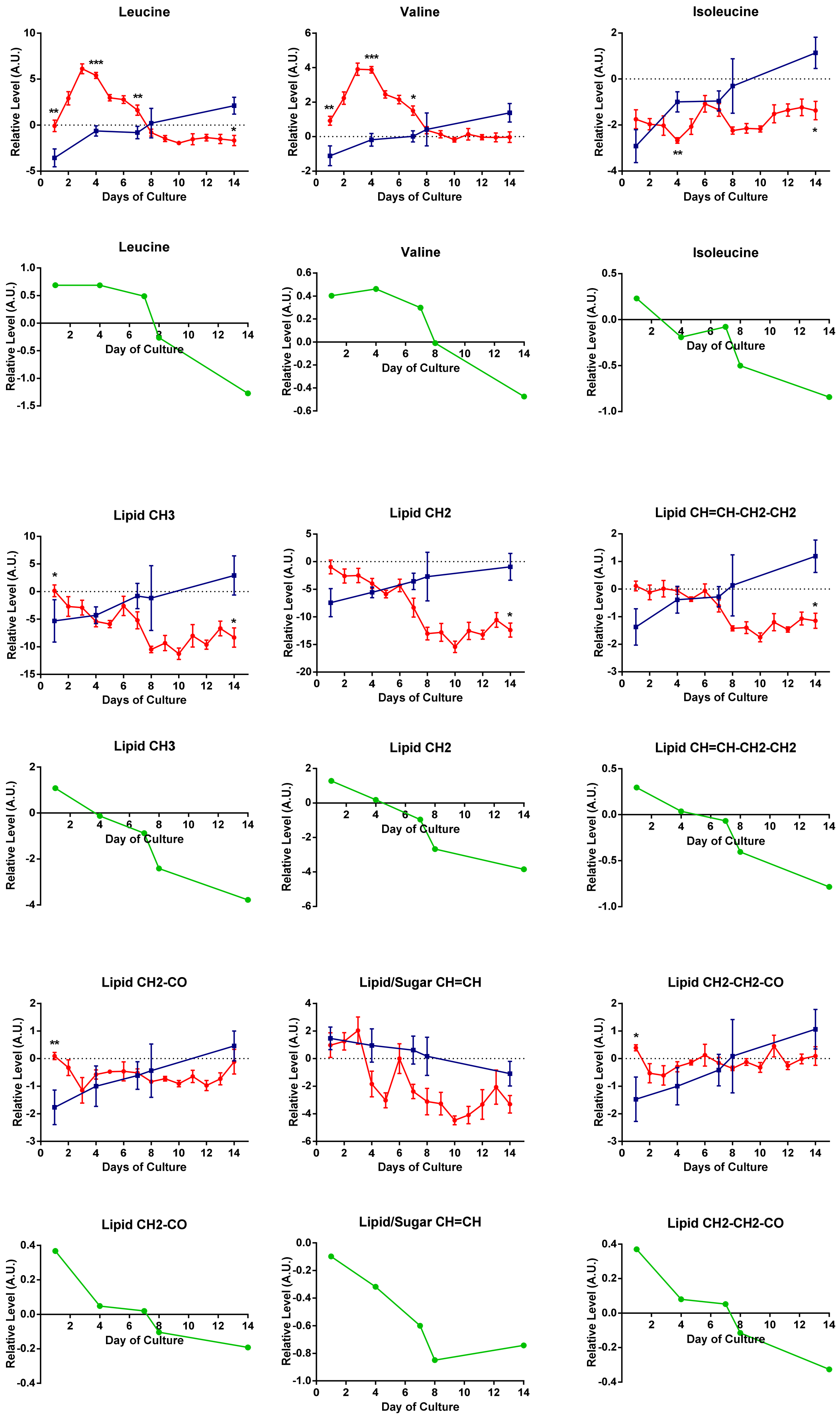
Leucine
Valine
Isoleucine
Relative Level (A.U.)
Days of Culture
Day of Culture
Lipid CH3
Lipid CH2
Lipid CH=CH-CH2-CH2
Lipid CH2-CO
Lipid/Sugar CH=CH
Lipid CH2-CH2-CO
